# Organic Carbon Controls Mercury Distribution and Storage in the Surface Soils of the Water-Level-Fluctuation Zone in the Three Gorges Reservoir Region, China

**DOI:** 10.3390/ijerph20043681

**Published:** 2023-02-19

**Authors:** Sihua Zhu, Caiyun Yang, Hong Chen, Yongmin Wang, Jieqin Li, Ruixi Zhang, Yu Yang, Cheng Zhang, Dingyong Wang

**Affiliations:** 1 Interdisciplinary Research Centre for Agriculture Green Development in Yangtze River Basin, College of Resources and Environment, Southwest University, Chongqing 400716, China; 2 Chongqing Key Laboratory of Agricultural Resources and Environment, College of Resources and Environment, Southwest University, Chongqing 400716, China

**Keywords:** mercury, soil organic carbon, distribution, storage, three gorges reservoir, water-level fluctuation zone

## Abstract

The particular condition of the water-level-fluctuation zone (WLFZ) in the Three Gorges Reservoir (TGR), the largest hydroelectric reservoir in China, raises great concerns about mercury (Hg) contamination and ecological risk. In addition, previous research found that soil organic carbon (SOC) plays an essential role in controlling Hg distribution and speciation. However, there is minimal information on the Hg storage distribution and their relationships with SOC in the WLFZ in TGR. This study investigated Hg distribution, storage, and their relationships with SOC in the surface soils in WLFZ. The results showed that the total Hg (THg) content in the surface soils ranged from 18.40 to 218.50 ng g^−1^, with an average value of 78.17 ± 41.92 ng g^−1^. About 89% of samples had THg content above the background value in Chongqing, showing specific enrichment of Hg in WLFZ due to contamination in the TGR. The surface soils have low SOC, with an average value of 8.10 ± 3.90 g kg^−1^. Moreover, THg content showed consistent distribution with the SOC in WLFZ, with a significantly positive correlation (*R* = 0.52, *p* < 0.01, *n* = 242). THg storage (201.82 ± 103.46 g ha^−1^) in the surface soils was also significantly positively correlated with the SOC storage (*R* = 0.47, *p* < 0.01, *n* = 242). The reduced SOC sequestration, due to the periodical alternative “flooding–draining” and frequent reclamation and utilization of WLFZ, decreased the Hg adsorption in soil. Those might result in the re-release of Hg into waters when WLFZ is flooded. Therefore, more attention should be directed towards Hg cycling and the consequent environmental risks in the TGR region.

## 1. Introduction

Mercury (Hg) is one of the most hazardous pollutants with potent toxicity [1,2]. Hg may be present in three chemical forms: elemental mercury (Hg^0^), divalent inorganic mercury (Hg^II^), and methylmercury (MeHg) [3]. It has received worldwide attention for decades due to its high toxicity, prevalent existence, and bioaccumulation through the food chain of its methylation product: MeHg [4,5]. The biogeochemical cycles of Hg can be influenced by soil organic carbon (SOC) through several aspects, including ligand binding [6] and redox processes [7]. Firstly, research shows that the adsorption/desorption of Hg species with sediment particles, which is mediated by the partitioning of associated organic ligands, is a primary driving force for the distribution of MeHg and Hg^Ⅱ^ [8]. Based on studies on sediments, and other high-SOC sites, it is generally accepted that SOC-rich soils show a greater capacity to immobilize Hg due to their binding and adsorption capacities [9,10,11]. Secondly, reducing Hg^II^ to Hg^0^ via SOC accounts for an important part of legacy Hg recycling back into the atmosphere [12,13,14]. Thus, untangling the relationships between SOC and Hg occurrence is necessary further to understand the Hg biogeochemical cycle in the terrestrial system.

A hydroelectric reservoir is a typical Hg-sensitive ecosystem resulting from the perturbation from water table changes [15]. In particular, the newly built reservoirs have become necessary points of focus for the observed Hg biogeochemistry. This specific ecosystem has been reported to provide an environment favorable for MeHg formation and the elevated MeHg in fish via food chain bioaccumulation [16,17,18]. As the flooded soil forms the anaerobic condition in the reservoir bottom, helping with the biotic Hg methylation involves a series of reducing bacteria clusters such as sulfate-reducing bacteria and methanogens [19]. Furthermore, accumulated SOC and plant debris in flooded soils provided sufficient energy for the growth of methylation bacteria and subsequent Hg methylation [20]. As a result, MeHg in fish and seafood was subsequently bioaccumulated along the food chain. Consequently, human communities with a traditionally high dietary intake of seafood should be subject to one of the most effective routes of exposure. Exposure to high levels of Hg has been reported to harm the brain, heart, kidneys, lungs, and immune system [2]. Therefore, for the reservoir system, understanding Hg biogeochemistry, especially the link between Hg distribution and environmental factors, is one of the most essential concerns because of the human health implications.

As the largest hydraulic engineering building in the world, the Three Gorges Reservoir (TGR) raised significant concerns about ecological and environmental issues since its impoundment [21,22,23,24]. The alternative wetting–drying pattern in the TGR is non-seasonal, which could cause significant changes in the environmental characteristics and soil physicochemical properties, significantly affecting the Hg behavior in the environment [25,26,27]. During the past few years, a series of studies were conducted systematically in TGR areas. Recent studies have discovered high levels of MeHg exposure in populations around the TGR that have a lot of fish in their diets, with hair Hg concentrations of up to 1.44 mg g^−1^ exceeding the US EPA criterion [26]. Most works mainly aimed at the aspects of bioaccumulation characteristics of THg and MeHg [27,28,29]. Additionally, the MeHg degree and distribution [30,31,32] and the effects of microorganisms [19] on Hg transfer. For example, previous studies have reported that the re-vegetation and root exudates are crucial in Hg cycling [33,34]. Some studies have found the relationship between elemental sulfur and MeHg [35,36] is significant. Additionally, the complicated associations between dissolved organic matter (DOM) and Hg [37] were explored and discussed in detail. However, there remains a critical knowledge gap that lacks a complete picture of the Hg content distribution in the surface soil at the WLFZ of the TGR areas. Regarding the crucial role of SOC in the Hg cycle, as mentioned, we expected the variations in the SOC in surface soils of WLFZ, which could be a key factor explaining the Hg storage in TGR areas.

To address the above concerns, we investigated the distribution and storage of Hg in the surface soil of WLFZ in the TGR. We analyzed and discussed the relationship between Hg contents and environmental factors, especially the role of SOC linking with Hg in WLFZ. In this study, our objectives were two-fold, including (1) understanding the Hg distributions in WLFZ from the view of a whole picture; and (2) validating our expectation and understanding the relationship between Hg storage and environmental factors, especially regarding the controlling role of SOC in WLFZ. Thus, this study will provide helpful information to further fulfill the knowledge pool of Hg biogeochemistry in the critical zones of the Earth system. The findings detailed here could also help to evaluate the environmental risks of Hg in WLFZ.

## 2. Materials and Methods

### 2.1. Study Area and Sample Collection

**Figure 1:**
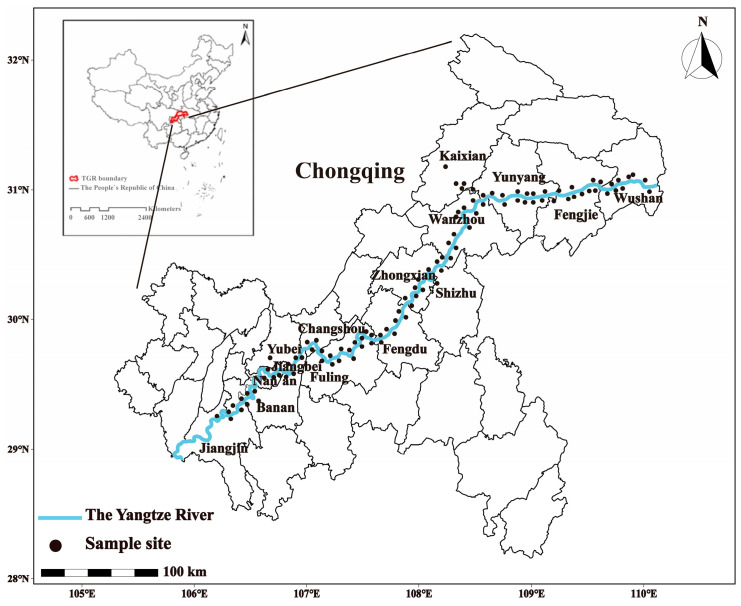
Sampling location of the study area.

The TGR, the largest hydroelectric reservoir in China, has a total area of flood landscapes of 630 km^2^. Among them, 350 km^2^ was a seasonally flooded WLFZ which is heavily influenced by human activities along the TGR. The TGR region is located in the 600 km section of the Yangtze River between the cities of Chongqing and Yichang. The area in this study is located in the Chongqing section of the TGR region (106°50′–110°50′ E, 29°16′–31°25′ N) between the Jiangjin District and Wushan County. The climate of the TGR area is subtropical monsoon, with an annual average temperature of 17.9 °C, sunshine duration of approximately 1630 h, average frost-free period of approximately 260 d, and annual precipitation of 1000–1800 mm [32]. Contrary to the natural wetting–drying aquatic system such as lakes and rivers, the anti-seasonal water level management controls the water level in TGR areas, changing from the 145 m a.s.l. (summer, called dry period) to 175 m a.s.l. (winter, called wet period). As a result of such non-seasonal fluctuations in the water-level, WLFZ is formed within a vertical height of 30 m (approximately a total area 350 km^2^) [38]. The main soil type in this area is purple soil and barren and agricultural lands (planted with corns by the local farmers from March to August) are the major land types in this area. From 2012–2013, during the two dry periods (i.e., from May to August) of each year, two sampling campaigns were conducted. In total, 15 sites were selected for soil sampling. The sampling sites cover Wushan, Fengjie, Yunyang, Kaixian, Wanzhou, Shizhu, Zhongxian, Fengdu, Feiling, Changshou, Banan, Nan’an, Jiangbei, Yubei, and Jiangjin (Figure 1), which are the administrative districts and counties in Chongqing city. Samples were harvested from the surface soil layer (0–20 cm). According to the sampling guide [39], one sampling plot was set for at least 10 m × 10 m. The five soil cores were obtained from the middle and four corners of each plot, which were further combined to form a composite sample. Overall, (*n* = 242) soil samples were collected in total. All soil samples were transported to the laboratory on ice in polyethylene plastic bags.

### 2.2. Analysis Method

The soil pH was measured by making soil slurry in a soil–water ratio of 1:2.5 (*w*/*v*). The mixture is stirred for 3 min and then filtered under gravity for a 30 min period. The filtrate’s pH represents the soil’s pH, and it is measured with a portable pH meter (ST300, OHRUS^®^, Cole-Parmer, Wertheim, Germany) [40]. The amorphous Fe oxide (Fe_o_) contents of the bulk soil samples were determined by ammonium oxalate buffer solution [41]. The soil cation exchange capacity (CEC) was measured using the sodium acetate method [42]. The physical and chemical properties of soil were presented in Table 1.

Hg in soil was determined via thermal decomposition atomic absorption spectrometry after gold amalgamation, using DMA–80 (Milestone, Italy). Two method blanks, three certified reference materials (CRMs), and 10% replicate samples were accompanied in each sample batch (up to 30 samples) for quality assurance (QA) and quality control (QC) of sample detection. The method detection limit (MDL) of Hg in soil was 0.009 ng g^−1^. The method blanks were lower than the detection limits in all cases. The SD of sample duplicates ranged from 0.28–10.5%. The recovery rate for CRMs in soil (GBW07406) ranged from 86–107%. A potassium dichromate external heating method measured the SOC. The soil bulk density was measured using the cutting-ring method [43].

The Hg storage in the soil of WLFZ in the TGR area is calculated as:

(1) $$S_{Hg}=C_{i}\times \rho_{i}\times h\times 10^{-1}$$

where $S_{Hg}$ is the total Hg storage in the soil (g ha^−1^); $C_{i}$ is the Hg content in the soil (ng g^−1^); $\rho_{i}$ is the soil bulk density (g cm^3^), and h is the soil thickness (cm).

The SOC storage in the soil is calculated as:

(2) $$SOC_{D}=SOC_{i}\times \rho_{i}\times h\times (1-\delta )\times 10^{-2}$$

where $SOC_{D}$ is the organic carbon storage in the soil (kg m^−2^); $SOC_{i}$ is the organic carbon content in soil (g kg^−1^); $\rho_{i}$ is the soil bulk density (g cm^−3^); h is the soil thickness (cm); and δ is the proportion of particles with a diameter larger than 2 mm.

### 2.3. Statistical Analysis

Data processing and analysis were conducted using SPSS 26.0 (IBM, Armonk, NY, USA) and the “ggplot2” package in the R version 4.2.2 [44]. Partial least square path modeling (PLS-PM) was used to confirm the correlations of soil chemical properties (pH, Fe_o_, CEC, SOC) with Hg distribution and storage. The partial least square (PLS) analysis was developed in 1960 to compensate for the limitations of multivariate normality and large sample sizes in the analysis using the existing linear structural relationships [45]. In particular, it has the ad-vantage of allowing analysis even when only a small number of samples are available. Path modeling (PM) is used to evaluate the validity and reliability of measurements and analyze the causal relationships among tested variables.

## 3. Results

### 3.1. Distribution of Hg in the Surface Soils

**Figure 2:**
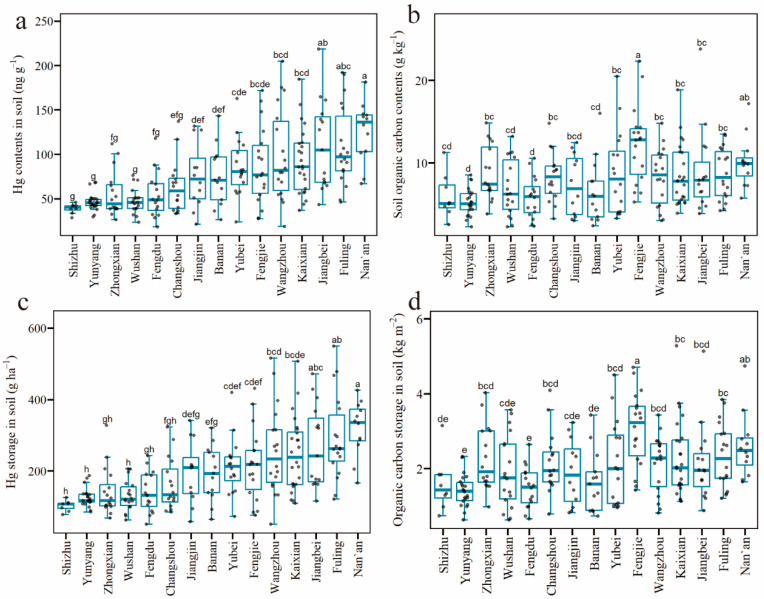
Distribution and storage of Hg and SOC in the soil of WLFZ, including (**a**) Hg contents; (**b**) SOC contents; (**c**) Hg storage; (**d**) SOC storage. Black dots represent values for individual sample sites. Different letters above each column represent the significant difference (*p* < 0.05).

The Hg contents in the surface soils of WLFZ in TGR ranged from 18.40 to 218.5 ng g^−1^, with a mean value of 78.17 ± 41.92 ng g^−1^. They varied significantly, with a variation coefficient of 53.63% (Table 2). Moreover, 89% of samples exceeded the background value in Chongqing (37.00 ng g^−1^) [46]. The average Hg content varied significantly and ranged from 38.9 to 125.5 ng g^−1^, which was 1.1–3.4 times higher than the background value in Chongqing [46]. The most considerable Hg content reached 218.5 ng g^−1^ (Figure 2a), 5.9 times the background value, indicating the particular Hg contamination in soils in the study area. The most serious contamination was found in Jiangbei, Fuling, and Nan’an, with the Hg contents in all the samplings sites above the background value. In contrast, although low content was found in Fengdu and Shizhu, 75% of samples had values above the background values.

### 3.2. Distribution of the Organic Carbon in the Surface Soil

The SOC content ranged from 2.28 to 23.79 g kg^−1^ (Table 3). The highest SOC content (12.20 ± 4.64 g kg^−1^) was found in Fengjie, and the lowest values (about 5.5 g kg^−1^) were found in Fengdu, Yunyang (Figure 2b). The SOC content in WLFZ in TGR was relatively low compared to that in other wetland areas in China [23,47]. The SOC (0–15 cm) in the Wanjiang wetland, located in the middle and lower reaches of the Yangtze River, was about 11.30–27.83 g kg^−1^, with a mean value of 17.00 ± 1.50 g kg^−1^ [23]. The SOC of the Dongting Lake wetland was above 40 g kg^−1^ [47].

### 3.3. Hg Storage in the Surface Soil

Hg storages in the soil in WLFZ in the TGR varied significantly, with a range value of 50.76–549.58 g ha^−1^ and a variation coefficient of 51.36% (Figure 2c). The average Hg storage was 201.83 ± 103.46 g ha^−1^ in the study area. The Hg storage showed different distribution from that of Hg content in a different area, which might be due to the different bulk densities. The highest Hg storage (318.43 ± 77.27 g ha^−1^) was found in Nan’an, and the lowest (102.82 g ha^−1^) was in Shizhu.

### 3.4. The SOC Storage in the Surface Soil

The estimated SOC storage of WLFZ in TGR was shown in Figure 2d. In the study area, the SOC storage ranged from 0.63 to 5.28 kg m^−2^, with an average value of 2.09 ± 0.95 kg m^−2^. The SOC storages varied with different districts/counties, with a variation coefficient of 14.56~48.26%. The highest SOC storage was found in Fengjie (3.06 ± 0.99 kg m^−2^), while the lowest was in Yunyang (1.41 ± 0.43 kg m^−2^).

### 3.5. The Physical and Chemical Properties of the Surface Soil

The physical and chemical properties of WLFZ in TGR was shown in Table 1. In the study area, the pH ranged from 4.73 to 8.54, with an average value of 7.54 ± 0.79. CEC and Fe_o_ in the soil in WLFZ in the TGR varied significantly, with a range value of 3.77–42.27 cmol kg^−1^ and 1067.50–6923.26 mg kg^−1^, respectively. The highest CEC (21.06 ± 9.95 cmol kg^−1^) was found in Fuling, and the lowest (10.81 ± 7.09 cmol kg^−1^) was found in Banan.

## 4. Discussion

### 4.1. Distribution and Storage of Hg in the Surface Soil

**Figure 3:**
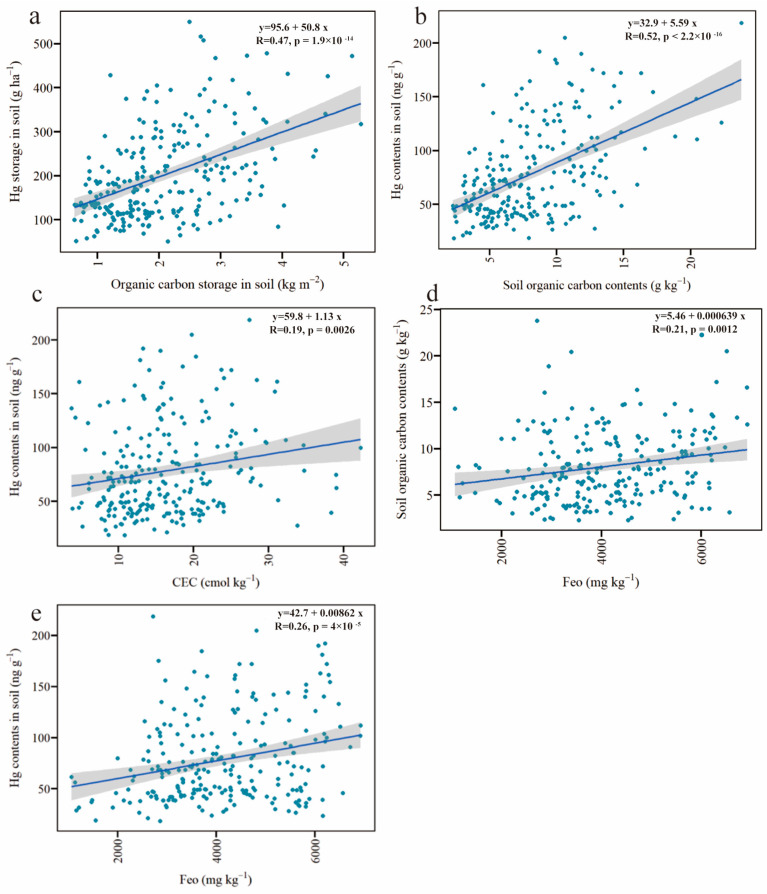
Person’s rank correlations (**a**) Hg storage in soil and SOC storage; (**b**) Hg contents and SOC contents; (**c**) Hg contents and CEC; (**d**) SOC contents and Fe_o_; (**e**) Hg contents and Fe_o_. Shown are the fitted regression lines and 95% confidence intervals, and blue dots represent values for individual sample sites.

Chongqing is a fast-developing industrial region in southwest China. Despite the control and management regulation of potentially toxic elements contamination in recent years, potential toxic elements, including Hg, might accumulate in the soils through atmospheric deposition due to the extensive production and emissions that occurred in the past. Those Hg might enter into the soils in TGR via rain wash and surface runoff during the draining period. The Hg storage here was much lower than previous measurements (102.9 ± 9.8 mg m^−2^) in the soil (0–40 cm) of Chongqing [48]. This might be due to the different sampling depths (0–20 cm), in which the Hg in surface soil could be reduced by washing off with waters and re-released into waters via the soil/water surface during the flooded period. Furthermore, these factors reduced the Hg storage but increased the environmental risk. In this study, the coefficient of variations (CV) of Hg contents was 53.63%, indicating large variations in spatial distributions for Hg. However, the correlation analysis found the Hg storage significantly positively correlated with the SOC in the soils in the WLFZ, with a Pearson correlation coefficient of 0.47 (*p* < 0.01, *n* = 242) (Figure 3a). This suggests the SOC storage in the soil greatly affected Hg storage regardless of the spatial variations of Hg distributions in WLFZ.

### 4.2. Distribution and Storage of SOC in the Surface Soils

Like other ecosystems, such as wetlands, WLFZ is substantially influenced by water levels. Thus, SOC turnover and accumulation due to oxic–anoxic degradation and plant debris inputs are active in WLFZ. The SOC content in WLFZ in TGR was relatively low compared to that in other wetland areas in China. The WLFZ in TGR represents a transition between aquatic and terrestrial systems with low soil gleization, vegetation/organisms, and the rapid decomposition of animals and plant residues. Under the periodical alternative wetting–drying condition in TGR, the hydrological characteristics, including water level fluctuation, velocity, and flow, varied significantly, resulting in a significant loss of soil as well as the residues of animals and plants, which decreased SOC content and carbon sequestration.

Previous studies found that the wetlands showed soil carbon sequestration and carbon sink [49], with the higher SOC storage in Wanjiang and Dongting lake wetlands discussed above. With wetland reclamation, the SOC content in the soil might decrease due to the reduced input of SOC and enhanced decomposition [23]. Here, the SOC storage was low compared to that in the two wetlands mentioned above. There are two potential explanations for this. One is that the majority of the WLFZ is utilized as agricultural soil by local peoples, resulting in a reduction in the SOC storage. A previous study has reported that conversion of non-cultivated land for agricultural purposes has substantially reduced global SOC stocks in upper soil layers, and the results are consistent [50]. The other possible explanation is that, as a new reservoir, WLFZ has limited soil carbon sequestration. Similar research has shown that reservoirs emit more carbon than they bury, challenging the current understanding that reservoirs are net carbon sinks [51].

### 4.3. Correlation between Hg Distribution, Storage, and SOC

The distribution of the Hg storage significantly positively correlated with the SOC storage (*R* = 0.47, *p* < 0.01, *n* = 242) (Figure 3a). One study found that Hg storage was significantly positively correlated with SOC and nitrogen in the soil in four forests in the Sierra Nevada [52]. This might be explained by the increased soil adsorption capacity, due to great surface litter in the forests, which enhanced the accumulation of atmospheric Hg deposited into the soil with the leaves. However, the surface litter was very limited in the WLFZ in the TGR under the alternative wetting–drying condition, resulting in low SOC content. Additionally, the great population and agricultural/industrial activities along the WLFZ could affect SOC storage. Therefore, the WLFZ was heavily influenced by human-induced disturbances and the periodical alternative wetting–drying condition. Those greatly affect carbon sequestration and Hg storage in WLFZ. The carbon sequestration and SOC storage in WLFZ were reduced due to the short formation time, frequent utilization, and reclamation. These factors might decrease the Hg absorption in the soil in WLFZ, resulting in the potential re-release of Hg into waters during the flooding period.

The Hg content in the soil of WLFZ in TGR was significantly positively correlated with that of SOC, with a Pearson correlation coefficient of 0.52 (*p* < 0.01, *n* = 242) (Figure 3b). The highest Hg content (218.5 ng g^−1^) and SOC (23.79 g kg^−1^) were found in Jiangbei, while the lowest Hg content (18.4 ng g^−1^) and SOC (2.34 g kg^−1^) was found in Fengdu. In addition, the SOC greatly affects the degradation of SOC and the soil’s CEC [53]. In this study, both the CEC and SOC significantly correlated with the Hg content, with correlation coefficients of 0.52 and 0.19 (*p* < 0.01, *n* = 242) (Figure 3b,c), respectively, implying that the SOC could influence Hg behavior in the soil as a function of the CEC. In addition, the Fe_o_ content in soils plays an important role in SOC stabilization, and the formation of organo-mineral complexes has been recognized as a critical mechanism [54]. Our results revealed that the SOC content in WLFZ in TGR significantly positively correlated with the Fe_o_ (*R* = 0.208, *p* = 0.001, *n* = 242) (Figure 3d). Additionally, the Fe_o_ and the Hg content showed a significantly positive correlation (*R* = 0.261, *p* < 0.001, *n* = 242) (Figure 3e), indicating Fe_o_ was a critical driving factor that significantly influenced the Hg and SOC storage in the soils of TGR. Furthermore, regarding the coupling between Fe_o_ and SOC for the SOC stabilization potential, the results (Figure 3a) indicate that in TGR, a higher stabilization degree of SOC shows greater capacities for holding Hg storage. This suggests that enhancing SOC stabilization might be a suitable strategy for offsetting the greenhouse emission feedback to global warming. Meanwhile, this methods helps to stabilize more Hg inputs from atmospheric deposits, resulting in less Hg losses from soils into nearby aquatic systems.

### 4.4. Partial Least Squares Path Modeling (PLS-PM) Analysis

**Figure 4:**
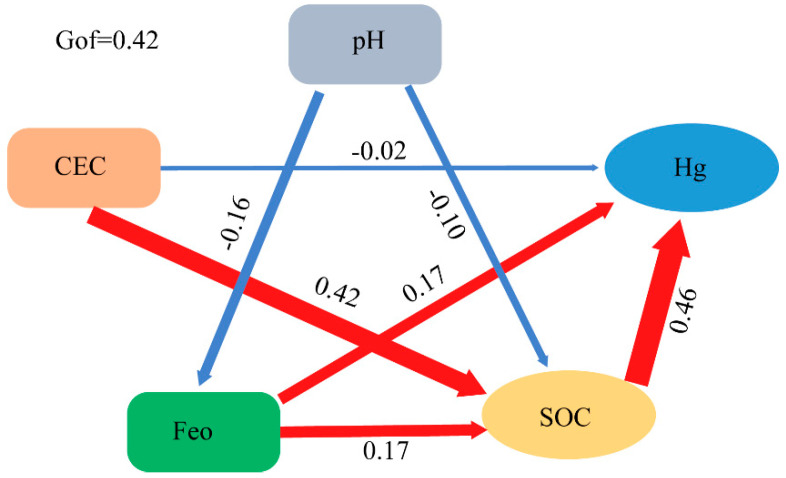
Cascading relationship of Hg with the physical and chemical properties of soil (pH, Fe_o_, CEC, and SOC) was shown by Partial least-squares path modeling (PLS-PM). Red and blue arrows indicate positive and negative pathways, respectively. The numbers on the arrow show standardized path coefficients, and the arrow widths are proportional to path coefficients.

Multivariate statistics analysis achieved via PLS-PM was selected to evaluate the influencing roles and relative contributions of pH, Fe_o_, CEC, and SOC on Hg distribution and storage. SOC includes SOC distribution and storage, and Hg is a collection of Hg distribution and storage. The standardized direct effects of pH, CEC, Fe_o_, and SOC on the Hg distribution and storage are shown in Figure 4. The goodness of fit (GoF) was 0.42, and SOC had the greatest direct positive effects (0.46) on Hg distribution and storage. This suggests that SOC distribution and storage determined the Hg distribution and storage, further supporting our correlation analysis results between Hg and SOC. Studies have reported a close link between SOC and Hg [55,56,57,58]. This study further demonstrates that SOC distribution and storage roles are predominant in controlling Hg distribution and storage.

## 5. Conclusions

Although we have explored mercury (Hg) biogeochemistry in the water-level fluctuation zone (WLFZ) of the Three Gorges Reservoir (TGR) areas to some degree during the past few years, this study provides a whole picture of Hg distribution and its relationship with environmental factors, especially soil organic carbon (SOC). We found that the Hg contents varied greatly in the surface soils of WLFZ in the TGR areas, and the average Hg content was significantly greater than the background value in Chongqing. Additionally, the spatial distributions of Hg contents and storage were linked with SOC content and amorphous Fe oxides (Fe_o_) in soils within significant correlations. These findings validated our expectation that SOC is crucial in controlling Hg storage in soils. Regarding the association between Fe oxides and SOC in regulating SOC stabilization, our results support SOC stabilization’s potential role in Hg storage in these areas. Based on the these extensive insights, destabilization of SOC resulted in harsher feedback of the soil carbon pool in relation to climate change, which also could elevate the releases and emissions of Hg that was stored previously. As a result, increases in environmental risks induced by Hg elevations could be expected in the context of the changing climate. Thus, policies and management for stabilizing SOC in the TGR areas will mitigate climate change feedback. It could also be beneficial to control Hg pools as sinks rather than sources. In the future, great attention and further study will be needed to examine the coupling between the carbon and mercury cycles in WLFZ in TGR areas.

## Figures and Tables

**Table 1:** The physical and chemical properties of soil.

County/District	pH	CEC (cmol kg^−1^)	Fe_o_ (mg kg^−1^)
Jiangjin	8.28 ± 0.75	15.73 ± 3.92	4202.42 ± 1042.86
Banan	7.74 ± 0.43	10.81 ± 7.09	4298.48 ± 1241.03
Nan’an	7.73 ± 0.43	14.16 ± 6.70	5426.34 ± 878.24
Jiangbei	7.88 ± 0.36	14.10 ± 9.46	3515.49 ± 609.66
Yubei	7.02 ± 0.90	17.64 ± 6.73	4524.21 ± 1194.03
Changshou	6.63 ± 1.25	11.39 ± 2.36	5153.01 ± 756.26
Fuling	7.18 ± 0.68	21.06 ± 9.95	5121.29 ± 1007.42
Fengdu	8.04 ± 0.19	13.74 ± 4.78	3546.34 ± 738.53
Shizhu	7.7 ± 0.19	13.75 ± 4.22	2591.38 ± 1012.06
Zhongxian	6.81 ±0.98	16.37 ± 5.10	3551.69 ± 1286.33
Wanzhou	7.43 ± 0.61	16.25 ± 6.27	4041.99 ± 1799.65
Kaixian	7.15 ± 0.61	16.70 ± 4.14	3222.15 ± 865.25
Yuyang	8.13 ± 0.69	16.78 ± 5.38	3618. 50 ± 801.57
Fengjie	7.50 ± 0.52	22.80 ± 8.72	4197.57 ± 1244.67
Wushan	8.19 ± 0.23	18.29 ± 8.71	4736.56 ± 1053.78

**Table 2:** Statistics analysis of Hg contents in the soil of WLFZ (ng g^−1^).

County/District	N	Mean	Min	Max	Median	SD	CV (%)
Jiangjin	12	76.0	21.2	131.6	72.2	37.1	48.8
Banan	13	75.2	26.5	143.2	70.9	33.5	44.5
Nan’an	12	125.5	66.8	181.2	136.5	33.8	27.0
Jiangbei	15	110.3	43.3	218.5	105.0	50.2	45.5
Yubei	16	85.4	23.8	162.7	80.6	32.6	38.2
Changshou	16	63.4	33.2	137.0	58.8	29.7	46.8
Fuling	18	108.9	46.1	192.1	97.2	85.6	45.4
Fengdu	16	54.4	18.4	118.0	48.8	25.8	47.4
Shizhu	8	38.9	28.6	45.8	40.3	5.5	14.2
Zhongxian	16	55.7	26.5	111.9	44.5	25.5	45.8
Wanzhou	18	99.3	18.8	204.8	82.0	52.6	53.0
Kaixian	24	92.2	36.8	184.5	86.2	37.3	40.5
Yuyang	22	46.8	29.6	68.3	45.4	10.0	21.4
Fengjie	18	86.9	27.5	172.0	77.2	43.9	50.5
Wushan	18	47.8	23.3	76.2	46.0	14.4	30.1

“N” means number of samples; “Min” means the minimum value; “Max” means the maximum value; “SD” means the standard deviation; “CV” means variable coefficient.

**Table 3:** Statistics analysis of SOC content in the soil of WLFZ (g kg^−1^).

County/District	N	Mean	Min	Max	Median	SD	CV (%)
Jiangjin	12	7.35	3.03	12.45	6.92	3.65	49.72
Banan	13	6.61	2.41	16.02	5.97	3.88	58.62
Nan’an	12	10.16	5.74	17.19	9.94	3.09	30.46
Jiangbei	15	8.92	3.89	23.79	7.92	5.10	57.15
Yubei	16	8.83	3.28	20.48	8.06	5.07	57.44
Changshou	16	8.34	3.25	14.81	8.35	2.97	35.62
Fuling	18	8.68	4.23	13.49	8.27	3.14	36.16
Fengdu	16	5.84	2.34	10.55	5.95	2.48	42.52
Shizhu	8	6.02	2.53	11.25	5.13	2.73	45.37
Zhongxian	16	8.82	3.83	14.83	7.46	3.31	37.49
Wanzhou	18	8.30	3.02	14.79	8.56	3.47	41.76
Kaixian	24	8.52	3.92	18.86	7.82	3.71	43.49
Yuyang	22	5.29	2.28	8.55	5.09	1.65	31.25
Fengjie	18	12.20	5.27	22.30	12.82	4.64	38.03
Wushan	18	6.98	2.29	13.22	6.25	3.42	49.00

“N” means number of samples; “Min” means the minimum value; “Max” means the maximum value; “SD” means the standard deviation; “CV” means variable coefficient.

## Data Availability

Data are available from the corresponding author upon reasonable request.

## Abbreviations

WLFZ	The water-level fluctuation zone
TGR	The Three Gorges Reservoir
Hg	Mercury
SOC	Soil organic carbon
THg	Total Hg
Hg^0^	Elemental mercury
Hg^II^	Divalent inorganic mercury
MeHg	Methylmercury
DOM	Dissolved organic matter
Fe_o_	Amorphous Fe oxide
CEC	Cation exchange capacity
PLS-PM	Partial least square path modeling

## References

[1] Driscoll C.T., Mason R.P., Chan H.M., Jacob D.J., Pirrone N. (2013). Mercury as a global pollutant: Sources, pathways, and effects. Environ. Sci. Technol..

[2] Kim K.-H., Kabir E., Jahan S.A. (2016). A review on the distribution of Hg in the environment and its human health impacts. J. Hazard. Mater..

[3] Beckers F., Rinklebe J. (2017). Cycling of mercury in the environment: Sources, fate, and human health implications: A review. Crit. Rev. Environ. Sci. Technol..

[4] Clayden M.G., Kidd K.A., Wyn B., Kirk J.L., Muir D.C.G., O’Driscoll N.J. (2013). Mercury biomagnification through food webs is affected by physical and chemical characteristics of lakes. Environ. Sci. Technol..

[5] Zhang W., Zhang X., Tian Y., Zhu Y., Tong Y., Li Y., Wang X. (2018). Risk assessment of total mercury and methylmercury in aquatic products from offshore farms in China. J. Hazard. Mater..

[6] Skyllberg U., Bloom P.R., Qian J., Lin C.-M., Bleam W.F. (2006). Complexation of mercury(II) in soil organic matter: EXAFS evidence for linear two-coordination with reduced sulfur groups. Environ. Sci. Technol..

[7] Jiang T., Wei S.-Q., Flanagan D., Li M.-J., Li X.-M., Wang Q., Luo C. (2014). Effect of abiotic factors on the mercury reduction process by humic acids in aqueous systems. Pedosphere.

[8] Hammerschmidt C.R., Fitzgerald W.F. (2004). Geochemical controls on the production and distribution of methylmercury in near-shore marine sediments. Environ. Sci. Technol..

[9] Padalkar P.P., Chakraborty P., Chennuri K., Jayachandran S., Sitlhou L., Nanajkar M., Tilvi S., Singh K. (2019). Molecular characteristics of sedimentary organic matter in controlling mercury (Hg) and elemental mercury (Hg^0^) distribution in tropical estuarine sediments. Sci. Total Environ..

[10] Teršič T., Biester H., Gosar M. (2014). Leaching of mercury from soils at extremely contaminated historical roasting sites (Idrija area, Slovenia). Geoderma.

[11] Teršič T., Gosar M. (2012). Comparison of elemental contents in earthworm cast and soil from a mercury-contaminated site (Idrija area, Slovenia). Sci. Total Environ..

[12] Gu B., Bian Y., Miller C.L., Dong W., Jiang X., Liang L. (2011). Mercury reduction and complexation by natural organic matter in anoxic environments. Proc. Natl. Acad. Sci. USA.

[13] TJiang T., Skyllberg U., Wei S., Wang D., Lu S., Jiang Z., Flanagan D.C. (2015). Modeling of the structure-specific kinetics of abiotic, dark reduction of Hg(II) complexed by O/N and S functional groups in humic acids while accounting for time-dependent structural rearrangement. Geochim. Cosmochim. Acta.

[14] Wei Z., Yu S., Adediran G.A., Tao J., Björn E. (2018). Mercury transformations in resuspended contaminated sediment controlled by redox conditions, chemical speciation and sources of organic matter. Geochim. Cosmochim. Acta.

[15] Hsu-Kim H., Eckley C.S., Achá D., Feng X., Gilmour C.C., Jonsson S., Mitchell C.P.J. (2018). Challenges and opportunities for managing aquatic mercury pollution in altered landscapes. Ambio.

[16] Johnston T.A., Bodaly R.A., Mathias J.A. (1991). Predicting fish mercury levels from physical characteristics of boreal reservoirs. Can. J. Fish. Aquat. Sci..

[17] Tremblay A., Lucotte M., Schetagne R. (1998). Total mercury and methylmercury accumulation in zooplankton of hydroelectric reservoirs in northern Québec (Canada). Sci. Total Environ..

[18] Friedl G., Wüest A. (2002). Disrupting biogeochemical cycles-consequences of damming. Aquat. Sci..

[19] Feng P., Xiang Y., Cao D., Li H., Wang L., Wang M., Jiang T., Wang Y., Wang D., Shen H. (2022). Occurrence of methylmercury in aerobic environments: Evidence of mercury bacterial methylation based on simulation experiments. J. Hazard. Mater..

[20] Yin D.L., Wang Y.M., Xiang Y.P., Xu Q.Q., Xie Q., Zhang C., Liu J., Wang D.Y. (2020). Production and migration of methylmercury in water-level- fluctuating zone of the Three Gorges Reservoir, China: Dual roles of flooding-tolerant perennial herb. J. Hazard. Mater..

[21] Wu W., Xie D., Liu H. (2019). Spatial variability of soil heavy metals in the three gorges area: Multivariate and geostatistical analyses. Environ. Monit. Assess..

[22] Ye C., Li S., Zhang Y., Zhang Q. (2011). Assessing soil heavy metal pollution in the water-level-fluctuation zone of the TGR, China. J. Hazard. Mater..

[23] Lin F., Li D., Pan G., Xu X., Zhang X., Chi C., Li Z. (2008). Organic carbon density of soil of wetland and its change after cultivation along the Yangtze River in Anhui province, China. Wetland Sci..

[24] Wang F., Zhang J. (2013). Mercury contamination in aquatic ecosystems under a changing environment: Implications for the TGR. Chin. Sci. Bull..

[25] Ye C., Li S., Zhang Y., Tong X., Zhang Q. (2013). Assessing heavy metal pollution in the water level fluctuation zone of China’s TGR using geochemical and soil microbial approaches. Environ. Monit. Assess..

[26] Cheng N., Xie Q., Fan Y.-F., Wang Y.-M., Zhang C., Wang D.-Y. (2018). Hair mercury concentrations in residents of fuling and Zhongxian in the three Gorges reservoir region and their influence factors. J. Environ. Sci..

[27] Xu Q., Zhao L., Wang Y., Xie Q., Yin D., Feng X., Wang D. (2018). Bioaccumulation characteristics of mercury in fish in the Three Gorges, China. Environ. Pollut..

[28] Xie Q., Wang Y., Li S., Zhang C., Tian X., Cheng N., Zhang Y., Wang D. (2021). Total mercury and methylmercury in human hair and food: Implications for the exposure and health risk to residents in the three gorges reservoir region, China. Environ. Pollut..

[29] Li J., Haffner G.D., Wang D., Zhang L., Li Y., Deng H., Drouillard K.G. (2018). Protein and lipid growth rates regulate bioaccumulation of PCBs and Hg in Bighead Carp (Hypophthalmichthys nobilis) and Silver Carp (Hypophthalmichthys molitrix) from the Three Gorges Reservoir, China. Environ. Pollut..

[30] Liu J., Wang D., Zhang J., Liem-Nguyen V., Huang R., Jiang T. (2020). Evaluation of hg methylation in the water-level-fluctuation zone of the three gorges reservoir region by using the mehg/hgt ratio. Ecotoxicol. Environ. Saf..

[31] Xiang Y.P., Du H.X., Shen H., Zhang C., Wang D.Y. (2014). Dynamics of total culturable bacteria and its relationship with methylmercury in the soils of the water level fluctuation zone of the Three Gorges Reservoir. Chin. Sci. Bull..

[32] Xiang Y.P., Wang Y.M., Zhang C., Shen H., Wang D.Y. (2018). Water level fluctuations influence microbial communities and mercury methylation in soils in the Three Gorges Reservoir, China. J. Environ. Sci..

[33] Yin D.L., Wang Y.M., Jiang T., Qin C.Q., Xiang Y.P., Chen Q.Y., Xue J.P., Wang D.Y. (2018). Methylmercury production in soil in the water-level-fluctuating zone of the Three Gorges Reservoir, China: The key role of low-molecular-weight organic acids. Environ. Pollut..

[34] Ma W., Li C., Zhang C., Wang D., Wang Y. (2023). Nutrients uptake and low molecular weight organic acids secretion in the rhizosphere of Cynodon dactylon facilitate mercury activation and migration. J. Hazard. Mater..

[35] Liu J., Jiang T., Huang R., Wang D., Zhang J., Qian S., Yin D., Chen H. (2017). A simulation study of inorganic sulfur cycling in the water level fluctuation zone of the Three Gorges Reservoir, China and the implications for mercury methylation. Chemosphere.

[36] Liu J., Jiang T., Wang F., Zhang J., Wang D., Huang R., Yin D., Liu Z., Wang J. (2018). Inorganic sulfur and mercury speciation in the water level fluctuation zone of the Three Gorges Reservoir, China: The role of inorganic reduced sulfur on mercury methylation. Environ. Pollut..

[37] Jiang T., Bravo A.G., Skyllberg U., Björn E., Wang D.Y., Yan H.Y., Green N.W. (2018). Influence of dissolved organic matter (DOM) characteristics on dissolved mercury (Hg) species composition in sediment porewater of lakes from southwest China. Water Res..

[38] Bao Y., Gao P., He X. (2015). The water-level fluctuation zone of Three Gorges Reservoir—A unique geomorphological unit. Earth Sci. Rev..

[39] Cheng J.-H., Tang X.-Y., Guan Z., Liu C. (2021). Occurrence of antibiotic resistome in farmland soils near phosphorus chemical industrial area. Sci. Total Environ..

[40] Liao J., Wen Z., Ru X., Chen J., Wu H., Wei C. (2016). Distribution and migration of heavy metals in soil and crops affected by acid mine drainage: Public health implications in Guangdong Province, China. Ecotoxicol. Environ. Saf..

[41] Shen Q., Demisie W., Zhang S., Zhang M. (2020). The association of heavy metals with iron oxides in the aggregates of naturally enriched soil. Bull. Environ. Contam. Toxicol..

[42] Meimaroglou N., Mouzakis C. (2019). Cation Exchange Capacity (CEC), texture, consistency and organic matter in soil assessment for earth construction: The case of earth mortars. Constr. Build. Mater..

[43] Dong J., Jiang Y., Lyu M., Cao C., Li X., Xiong X., Lin W., Yang Z., Chen G., Yang Y. (2023). Drought Changes the Trade-Off Strategy of Root and Arbuscular Mycorrhizal Fungi Growth in a Subtropical Chinese Fir Plantation. Forests.

[44] R Core Team (2022). R: A Language and Environment for Statistical Computing.

[45] Nam C., Kwon D.S., Lee M. (2014). Is technical expert compensated for the education on finance and management?. J. Digit. Converg..

[46] Chen N., Lai W., Xu M., Zheng C. (1982). Background values of 11 elements in soil of Chongqing in China. Chongqing Environ Prot..

[47] Zhang W., Peng P., Tong C., Wang X., Wu J. (2005). Characteristics of distribution and composition of organic carbon in Dongting lake floodplain. Environ. Sci..

[48] Zhou J., Wang Z., Sun T., Zhang H., Zhang X. (2016). Mercury in terrestrial forested systems with highly elevated mercury deposition in southwestern China: The risk to insects and potential release from wildfires. Environ. Pollut..

[49] Brix H., Sorrel B.K., Lorenzen B. (2001). Are phragimates dominated wetlands a net source or net sink of greenhouse gases. Aquat. Bot..

[50] Emde D., Hannam K.D., Most I., Nelson L.M., Jones M.D. (2021). Soil organic carbon in irrigated agricultural systems: A meta-analysis. Global Chang. Biol..

[51] Keller P.S., Marcé R., Obrador B., Koschorreck M. (2021). Global carbon budget of reservoirs is overturned by the quantification of drawdown areas. Nat. Geosci..

[52] Obrist D., Johnson D.W., Lindberg S.E. (2009). Mercury concentrations and pools in four Sierra Nevada forest sites, and relationships to organic carbon and nitrogen. Biogeosciences.

[53] Bronick C.J., Lal R. (2005). Soil structure and management: A review. Geoderma.

[54] Li F., Liang N., Zhang P., Xu Y., Chang Z., Wu M., Duan W., Steinberg C.E.W., Pan B. (2018). Protection of Extractable Lipid and Lignin: Differences in Undisturbed and Cultivated Soils Detected by Molecular Markers. Chemosphere.

[55] Sanei H., Outridge P.M., Stern G.A., Macdonald R.W. (2014). Classification of mercury–labile organic matter relationships in lake sediments. Chem. Geol..

[56] He M., Tian L., Braaten H.F.V., Wu Q., Luo J., Cai L.-M., Meng J.-H., Lin Y. (2019). Mercury–Organic Matter Interactions in Soils and Sediments: Angel or Devil?. Bull. Environ. Contam. Toxicol..

[57] Chakraborty P., Sarkar A., Vudamala K., Naik R., Nath B.N. (2015). Organic matter—A key factor in controlling mercury distribution in estuarine sediment. Mar. Chem..

[58] Wang Y., Liu J., Liem-Nguyen V., Tian S., Zhang S., Wang D., Jiang T. (2021). Binding strength of mercury (ii) to different dissolved organic matter: The roles of dom properties and sources. Sci. Total Environ..

